# Enhancing CO_2_ Adsorption on MgO: Insights into Dopant Selection and Mechanistic Pathways

**DOI:** 10.3390/biomimetics10010009

**Published:** 2024-12-27

**Authors:** Shunnian Wu, W. P. Cathie Lee, Hashan N. Thenuwara, Xu Li, Ping Wu

**Affiliations:** 1 Entropic Interface Group, Engineering Product Development, Singapore University of Technology and Design, 8 Somapah Road, Singapore 487372, Singapore; 2 Institute of Materials Research and Engineering, Agency for Science, Technology and Research (A*STAR), Fusionopolis Way, Innovis, Singapore 138634, Singapore

**Keywords:** MgO, CO_2_ adsorption, doping, charge donation, uneven electron distribution

## Abstract

Inspired by our recent success in designing CO_2_-phobic and CO_2_-philic domains on nano-MgO for effective CO_2_ adsorption, our ongoing efforts focus on incorporating dopants into pristine MgO to further enhance its CO_2_ adsorption capabilities. However, a clear set of guidelines for dopant selection and a holistic understanding of the underlying mechanisms is still lacking. In our investigation, we combined first-principles calculations with experimental approaches to explore the crystal and electronic structural changes in MgO doped with high-valence elements (Al, C, Si, and Ti) and their interactions with CO_2_. Our findings unveiled two distinct mechanisms for CO_2_ capture: Ti-driven catalytic CO_2_ decomposition and CO_2_ polarization induced by Al, C, and Si. Ti doping induced outward Ti atom displacement and structural distortion, facilitating CO_2_ dissociation, whereas C doping substantially bolstered the electron donation capacity and CO_2_ adsorption energy. Pristine and C-doped MgO engaged CO_2_ through surface O atoms, while Al-, Si-, and Ti-doped MgO predominantly relied on dopant–O atom interactions. Our comprehensive research, integrating computational modeling and experimental work supported by scanning electron microscopy and thermal gravimetric analysis, confirmed the superior CO_2_ adsorption capabilities of C-doped MgO. This yielded profound insights into the mechanisms and principles that govern dopant selection and design.

## 1. Introduction

The rise in greenhouse gas emissions has greatly accelerated global climate change. Among these emissions, carbon dioxide (CO_2_), primarily generated from fossil fuel combustion, constitutes approximately 70% of the total greenhouse gases released into the atmosphere [1]. Flue gases, which are released during industrial and power generation processes, are a major source of CO_2_. The efficient capture of CO_2_ from flue gases offers a promising strategy for mitigating the rise in greenhouse gas emissions. Among the various approaches, carbon capture and storage (CCS) technology stands out as an effective solution to address the challenges of climate change. CCS encompasses an integrated process involving CO_2_ capture, transportation, storage, and utilization. CO_2_ capture methods can be broadly categorized into adsorption, absorption, membrane separation, and cryogenic distillation techniques [2]. Among these, adsorption has been regarded as a competitive solution owing to its simple process, mild operating conditions, large operating flexibility, wide operating temperature range, lack of corrosion and fouling, stable performance, and low operating cost [2].

MgO has attracted extensive attention to be employed as a solid CO_2_ adsorbent to reduce CO_2_ due to its rich sources and low cost as well as its high theoretical CO_2_ adsorption capacity [3]. It is well known that pristine MgO exhibits a relatively low CO_2_ adsorption capacity, with commercial MgO adsorbing only 1.9 mg/g at 200 °C [4] and another study reporting 8.8 mg/g at 50 °C [5]. The limited adsorption capacity of unmodified MgO is one of the main reasons why structural modification and doping have attracted continuous research interest as a method to improve MgO’s performance. Inspired by the interwoven or intermittent water harvesting mechanism of the Namib desert beetles [6], which features alternating regions—a water capture region with high water adsorption and a water transport region with low water adsorption—our research group innovatively developed interwoven CO_2_-phobic and CO_2_-philic domains on electrospun nano-MgO, leading to a significant improvement in CO_2_ adsorption as high as 41 mg/g [7]. Another example of structural modification was demonstrated when Sun et al. decomposed Mg(OH)_2_ to produce MgO with a CO_2_ adsorption performance of 33 mg/g [8]. Additionally, many dopants have been introduced to MgO using different synthesis approaches. MgO containing 15 wt% La_2_O_3_ shows a comparatively high CO_2_ capacity of 26 mg/g and excellent cyclic stability [9]. The formatted carbonate layers were claimed not to shelter the abundant pores in the inside or outside of the hollow microspheres due to the increased oxygen vacancy with the addition of La [9]. Cs-doped MgO reports a CO_2_ sorption capacity of 76.6 mg/g at an adsorption temperature of 300 °C and 32.2 mg/g at 235 °C. It is speculated that the Cs doping should be increased so that a greater portion of adsorbent can react with CO_2_ to form a mixed Mg−Cs carbonate phase at a lower activation energy [10]. MgO that was introduced with elemental Fe and Ni by a ball-milling process obtained CO_2_ adsorption capacities of 36.3 and 60.9 mg/g, respectively [11]. This is significantly higher compared with the CO_2_ adsorption capacity of 15.7 mg/g for the undoped MgO synthesized by a solution–combustion process. Ni doping shows a more favorable influence on CO_2_ adsorption than Fe doping, since it brings about a narrower particle size distribution, which generates more active sites for CO_2_ adsorption. The incorporation of Al is claimed to fully expose the alkaline binding sites on the MgO surface, thereby promoting the adsorption capacity of CO_2_ and achieving an enhanced CO_2_ adsorption capacity of 34.1 mg/g [12]. This significant improvement in CO_2_ adsorption can be attributed to the excellent dispersity of MgO particles and the shortened diffusion path for CO_2_ molecules. A synergistic interaction between co-doped Al and C has been shown to produce highly unsaturated basic O^2−^ sites, enhancing CO_2_ adsorption [13]. Al-C-doped MgO instantly adsorbs 34.6 mg/g CO_2_ in the harsh environment of 200 °C. MgO doped with Cu [14], Zn [4], and Zr [15,16] achieved high sorption capacity of 34.6 mg/g [14], 33.3 mg/g [4], and 51.6 mg/g [15], respectively. It is proposed that the metal incorporation increases the surface defects of magnesia nanoparticles and generates abundant basic sites.

CO_2_ adsorption can be accomplished by dispersion forces or by carbonate formation via reaction with the five-coordinated O^2−^ ion on the exposed MgO surface. A theoretical study uncovered that no carboxylate species can form on the (100) surface of MgO in the absence of point defects [17]. On the other hand, doping with alkaline metals (Li, Na, K, and Rb) brings about a large increase in CO_2_ adsorption energies [18]. Therefore, all alkali metals enhance CO_2_ adsorption. Additionally, Sr doping on this enhanced surface makes the system more effective as a reversible CO_2_ adsorbent by lowering the adsorption energy of CO_2_ [19]. When doped with Al or any metal that is capable of acting as an electron donor, the formation of carboxylate species (CO_2_^−^) is favored. This is because the CO_2_ in the physisorbed state is unstable and will spontaneously transform into the carboxylate complex in the presence of an excess electron [17]. Although the doping of the MgO surface with Ni is found to have a weak effect on the energies of CO_2_ chemisorption [20], the introduction of intermetallics, NiM (M = Mn, Fe, Co, and Cu), on the MgO surface shows a stronger adsorption of CO_2_, which depends on the binding energies of NiM on MgO [20]. In the case of monometallic doping, the average CO_2_ adsorption energies of doped MgO increase in the order of Ca, Fe, and Al doping corresponding to the relevant chemical valence (+2 for Ca, +2/+3 for Fe, and +3 for Al) [21]. Strong attractive forces of CO_2_ to dopant atoms are mostly responsible for the increased adsorption strengths, especially for Al atoms. The CO_2_ binding interaction with the basic O sites in the doped MgO surface is enhanced compared with the undoped MgO surface. It is suggested that Al-doped MgO is more suitable as a CO_2_ adsorbent than Ca/Fe-doped MgO [22].

Despite ongoing experimental and theoretical research on doping strategies to enhance CO_2_ adsorption, clear guidelines for dopant selection and the underlying improvement mechanisms remain lacking. In the present study, CO_2_ adsorption on a pristine MgO (100) surface and a MgO (100) surface doped with selected dopants was evaluated. Density of states (DOS) and density-derived electrostatic chemical (DDEC) analyses [23,24,25,26] were conducted to characterize the doping effects and exploit the mechanism.

## 2. Materials and Methods

### 2.1. Theoretical Simulation of CO_2_ Adsorption

The Vienna ab initio simulation package (VASP) [27] was employed to carry out the first-principles calculations with the Perdew–Burke–Ernzerhof (PBE) generalized gradient approximation (GGA) exchange-correlation functional [28]. A projector augmented wave (PAW) method [29,30] was applied as a plane-wave basis set to describe the electron–core interaction. A kinetic energy cutoff of 500 eV was applied for the plane-wave expansion. The van der Waals interactions were accounted for using the DFT+D3 correction method [31]. The total energy convergence was set as 1.0 × 10^−6^ eV, and the forces on each individual atom were minimized to be smaller than 0.01 eV/Å for geometry optimization and total energy calculations. The value for smearing was fixed at 0.01 eV. A Monkhorst−Pack [32] K-points mesh was adopted for sampling the Brillouin zone, and the K-points number (NK) was adjusted to keep NK × L (L is the lattice constant) being about 25 Å for structural relaxations and 45 Å for electronic calculations, respectively.

The MgO crystalline structure obtained in a previous study [33] is used in this work. Its crystalline structure was cleaved in the most stable (001) direction [34] to investigate its ability to achieve CO_2_ adsorption. The MgO slabs were composed of six layers from the 3 × 3 expansion of the MgO unit cell. The lowest 3 layers remained fixed in their bulk locations while the top 3 layers and CO_2_ molecule were free to move. The doping effect on adsorption behavior was investigated by substituting one Mg atom with a dopant atom on the MgO (100) subsurface. One CO_2_ molecule was placed next to the dopant to evaluate its effect. To ensure that the interaction force between the layer planes was minimal, a 20 Å vacuum thickness was maintained between them. The CO_2_ adsorption energy $E_{ad}$ is defined as $E_{ad}=E_{surface+CO_{2}}-E_{CO_{2}}-E_{surface}$, where $E_{surface+CO_{2}}$ is the total energy of the system with the CO_2_ molecule, $E_{CO_{2}}$ is the energy of the CO_2_ molecule, and $E_{surface}$ is the total energy of the system. A lower value for $E_{ad}$ indicates a stronger adsorption of CO_2_ on the surface. The DDEC6 analysis was calculated using the Chargemol package [35]. The simulation procedure [36] is outlined in Table S1. 

### 2.2. Materials

Magnesium acetate tetrahydrate (Mg(CH_3_COO)_2_·4H_2_O) (ACS reagent, ≥98%) and poly(vinyl alcohol) (Mw 30,000–50,000, 87–90% hydrolyzed) were purchased from Sigma Aldrich (Merck Pte. Ltd., Singapore). Deionized water was obtained from Neptec Halios lab water system.

### 2.3. Preparation of MgO and C-Doped MgO Nanoparticles

MgO nanoparticles and C-doped MgO nanoparticles were synthesized using the spray-drying method. Initially, 4 g of magnesium acetate tetrahydrate were dissolved in deionized water to prepare a 40 g/L precursor solution. For the synthesis of C-doped MgO, additional polyvinyl alcohol was introduced into the solution at a 1:10 molar ratio to magnesium acetate tetrahydrate. This solution was fed into a laboratory scale mini spray dryer machine (BUCHI Mini Spray Dryer B-290, BUCHI, Flawil, Switzerland) at a flow rate of 10 mL/min and an evaporation temperature of 220 °C. The powder collected from the spray dryer was calcined in a muffle furnace (Carbolite ELF, Carbolite Gero, Derbyshire, UK) at 350 °C for 1 h, with a ramping rate of 2 °C/min. After natural cooling to room temperature, the calcined material was ground into fine nanopowder using a mortar and pestle, yielding the final nanoparticles.

### 2.4. Morphology Characterization and CO_2_ Adsorption Measurement

Surface morphology was characterized using FESEM instrument (JEOL JSM-7600F, Jeol, Tokyo, Japan). X-ray diffraction (XRD) analysis was performed using a Bruker D8 Advance X-ray diffractometer (Bruker, Karlsdorf, Germany) with Cu-Kα radiation (λ = 1.54 Å), operating at 40 kV and 25 mA. CHNS analysis was conducted using a Thermo Scientific Flash EA 1112 Series CHNS-O analyzer (Thermo Scientific, Waltham, MA, USA) to determine the carbon content of the doped sample. CO_2_ capture capacity was measured by thermogravimetric analysis (TGA) using TGA Q50 analyzer (TA Instruments, New Castle, DE, USA). For TGA, initially, samples were preheated at 150 °C for 60 min under high-purity N_2_ gas flow (40 mL/min) to remove preabsorbed moisture and other contaminants from the atmosphere during storage. Subsequently, high-purity CO_2_ gas was introduced at 30 °C at a rate of 40 mL/min for designated time period. The CO_2_ capture capacity was calculated from the weight gain of the sample during CO_2_ gas feed.

## 3. Results and Discussion

### 3.1. Structure of Pristine and Doped MgO Surface

**Figure 1:**
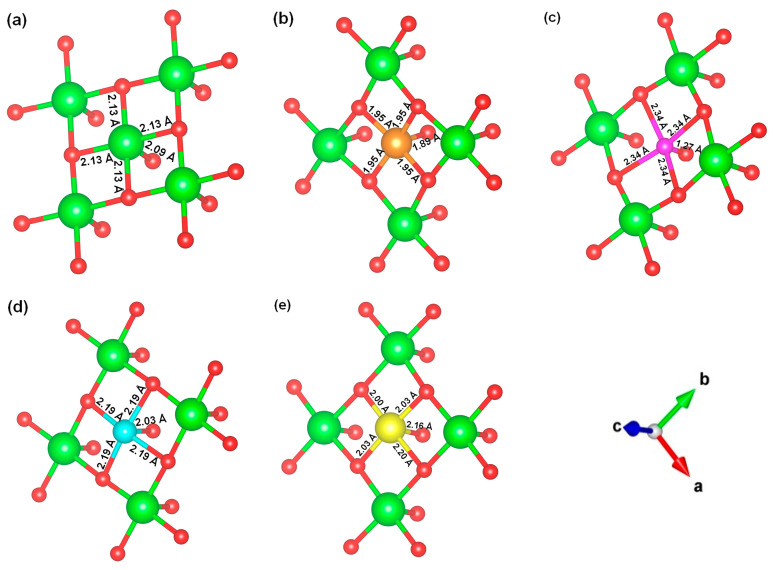
Optimized structure of MgO surface (**a**) and MgO surface doped with Al (**b**), C (**c**), Si (**d**), and Ti (**e**). Red, green, coral, magenta, cyan, and yellow balls represent O, Mg, Al, C, Si, and Ti atoms, respectively.

We replaced one Mg atom with one dopant atom, i.e., Al, C, Si, or Ti on the MgO surface in this work. Figure 1 shows the optimized structures of the doped MgO surfaces in comparison with the pristine MgO surface. The Mg atoms on the surface are five-coordinated with four equivalent surface O atoms to form Mg-O bonds of 2.13 Å and one slightly shorter vertical Mg-O bond of 2.09 Å. Al doping generates shorter Al-O bonds, indicating stronger interaction. The doped C shifts deeper to the layer next to the surface, with a significantly shorter C-O bond of 1.27 Å, which is close to the C=O bond of 1.28 Å in a carbonate group [37]. Contrarily, the C-O bond with the four surface O atoms is weak, and the bond length is 2.34 Å. Therefore, the C atom deviates from the original lattice site, and a defect is generated. Doped Si seems to move slightly inward to the neighboring layer, which may slightly stretch the four equivalent surface Si-O bonds. The dopant Ti shows exceptional outward movement, and the bond length of Ti with the O in the neighboring layer increases by 0.09 Å. Moreover, its bonds with the four surface O atoms are not equivalent, indicating torture of the structure. This may be attributed to the bonding using 3d orbitals of the transition metal atoms.

Table 1 lists the calculated doping energy $E_{f}$ required to dope the atom in the substitutional site using the following equation:

(1) $$E_{f}=E_{MgO-X}-E_{MgO}+E_{Mg}-E_{X}$$

where $E_{MgO}$ and $E_{MgO-X}$ are the energy of the parent MgO surface and doped MgO surface, respectively; $E_{Mg}$ and $E_{X}$ are the calculated energies of the element Mg and dopants, respectively, in their most stable structure [38], i.e., Mg of hexagonal P6_3_/mmc space group, Si of diamond Fd$\bar{3}$m1, C of hexagonal P6_3_/mmc structure, Al of hexagonal P6_3_/mmc structure, Ti of hexagonal close-packed P6_3_/mmc. It is observed that the $E_{f}$ for Al doping is the smallest, while the $E_{f}$ for C doping is the largest. A larger $E_{f}$ value indicates more energy input is required to generate the dopant site in the structure; therefore, it is tougher to obtain the doped structure. This may explain the abundance of experiments on Al-doped MgO and the lack of experiments on the other doped MgO surfaces.

The charge donation capacity is obtained by deduction of the calculated net atomic charge with the DDEC6 analysis from its valence electrons. It is observed that each Mg donates 0.54 electrons in pristine MgO, which is the least compared with all the dopants studied. Al donates more than double the electrons to MgO surfaces compared with Mg. However, less than half of its three valence electrons take part in direct bonding with neighboring O atoms. A significant increase in charge donation capacity is observed in C and Si, which donate 3.70 and 3.37 electrons to the structure, respectively. This implies that nearly all their four valence electrons are involved in bonding, which suggests about a 3.7+ and 3.4+ nominal valence state for C and Si, respectively. Ti shows about a 2.5+ nominal valence state, giving much larger charge transfer capacity than Mg. Niedermaier et al. also reported that while Co substitutes the host ions in the cationic sublattice of MgO, Co^2+^ ions keep their divalent valence state [39]. The increased electron transfer capacity of the dopant in doped MgO can transfer more electrons to the introduced CO_2_ molecules, thus improving its CO_2_ adsorption behavior [33].

### 3.2. Simulated CO_2_ Adsorption on Pristine and Doped MgO

Typical adsorption sites [17,40,41], i.e., the on-top site of O, on-top site of the dopant, bridging site of the Mg-O bond, bridging site of the dopant–O bond, and hole site, as shown in Figure S1, have been evaluated in our preliminary work. CO_2_ energetically prefers to adsorb on the on-top site of O on both pristine and C-doped MgO surfaces. Kim et al. [42] also reported that CO_2_ is strongly adsorbed at the on-top site of the oxygen on the MgO (100) surface. CO_2_ energetically prefers the hole site on Al-, Si-, and Ti-doped MgO surfaces. This is consistent with Lv et al.’s report that CO_2_ is strongly adsorbed at the hole sites on the Al-doped MgO (100) surface [43]. Table 2 summarizes the initial and optimized configurations for MgO with the adsorbate molecule CO_2_. The adsorbed CO_2_ molecule has changed from a linear shape to a bent structure in the pristine MgO and MgO doped with Al, C, and Si. Direct bonding of the O of the CO_2_ molecule with the dopant is observed in Al-, Si-, and Ti-doped MgO. The bonding stabilizes the adsorbed CO_2_ molecules, which explains the fact that CO_2_ prefers hollow sites in these MgO surfaces. However, due to the small size of the C cation, the bonding of O of CO_2_ with the dopant C does not occur; therefore, CO_2_ prefers the on-top site of O on the C-doped MgO surface.

Table 3 lists the calculated CO_2_ adsorption energies for pristine and doped MgO surfaces. All dopants negatively increase the CO_2_ adsorption energy. This indicates enhanced CO_2_ adsorption. Al doping negatively increases the adsorption energy by 37.5%, which is consistent with the significantly improved CO_2_ adsorption observed in experiments [12]. C, Si, and Ti doping dramatically increases the adsorption energy, indicating significantly enhanced affinity for CO_2_ molecules. This implies that MgO doped with C, Si, and Ti should exhibit noticeably improved CO_2_ adsorption performance. The distances of CO_2_ molecules from the surfaces and their structural parameters are listed in Table 3. The distances between CO_2_ and the surface ranged from 1.42 Å to 1.83 Å. C atoms of the adsorbed CO_2_ molecules are nearer to the pristine and C-doped MgO surfaces, whereas O atoms of the adsorbed CO_2_ molecules are nearer to the Al-, Si-, and Ti-doped MgO surfaces. It is noticed that the distance between CO_2_ and the surface is generally smaller for CO_2_ adsorption at the on-top site with an O preference.

The adsorption of CO_2_ can be described as a two-step process [44]: First, CO_2_ undergoes a deformation from its linear gas phase structure into a bent CO_2_ fragment; the deformation may be caused by the coordination of one carbon dioxide oxygen atom to the metal center. Second, the bent CO_2_ fragment binds to the substrate, which drives the adsorption process and reflects the adsorption capability of the substrate. Our calculated O–C–O bond angle (<OCO) of CO_2_ ranged from 116.37° to 134.00°, and the C–O bond length (d_C-O_) ranged from 1.24 Å to 1.42 Å, which are dramatically different from those of free CO_2_ molecules. The C=O bond length in carbon dioxide is 1.16 Å [37]. In its normal state, the C-O bond length is just 1.43 Å; however, the bond length can be stretched to 1.54 Å [37]. Therefore, the bond length shown in Table 3 indicates that the bonding in CO_2_ has not changed to a single C-O bond. Bond lengths of C=O bonds are around 1.23 Å in carbonyl groups and 1.28 Å in carbonate group anions [37]. Therefore, CO_2_ adsorption on the doped MgO surface may be due to chemisorption through the formation of a carbonate structure. The Ti-doped MgO surface shows the strongest polarization toward the CO_2_ molecule, eventually breaking its O=C=O structure, which may be attributed to the 3d orbitals of the Ti dopant. This suggests an additional benefit of the CO_2_ reduction reaction. This validates the effectiveness of using charge transfer to overcome the CO_2_ activation barrier during the initial stage of the CO_2_ reduction reaction, as indicated in Table 2. In the case of MOF-based nanomaterials, CO_2_ has been reduced by Ni, Co, Zn, and Cu using this strategy [45].

### 3.3. Density of States and Charge Transfer Analysis

**Figure 2:**
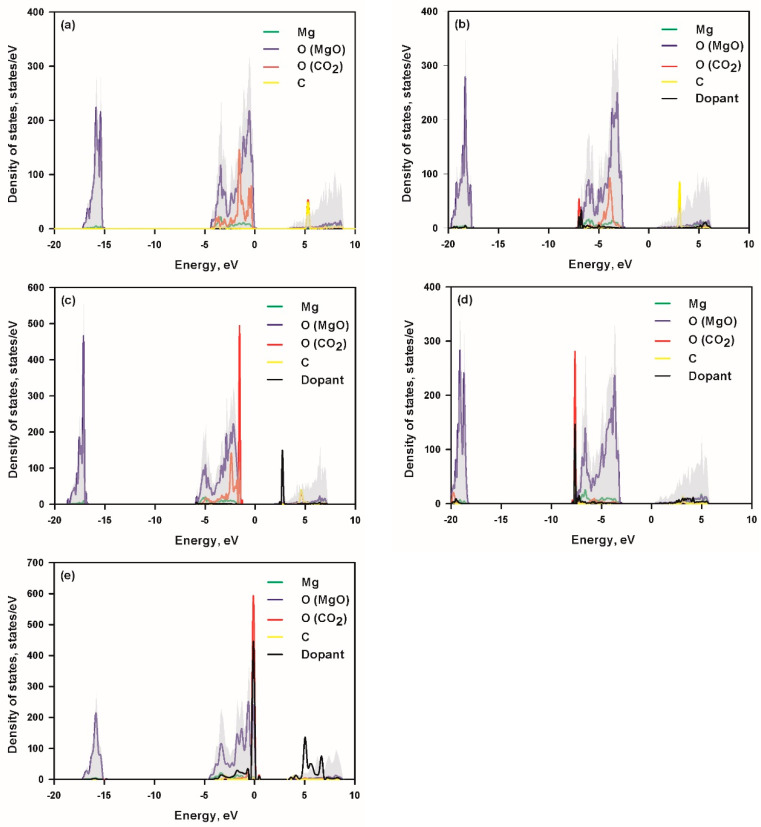
Total and projected density of states of MgO (**a**) and MgO doped with Al (**b**), C (**c**), Si (**d**), and Ti (**e**) after CO_2_ adsorption. O (MgO) and O (CO_2_) denote O from MgO and O from CO_2_, respectively.

The density of states (DOS) analysis [33] was carried out and is shown in Figure 2 to reveal the interaction of the CO_2_ molecule with pristine and doped MgO (100) surfaces. In order to achieve clear observation, the projected density of states (PDOS) of the dopant is magnified 25-fold; the PDOS of C and O of CO_2_ are magnified 25-fold for pristine MgO as well as Al- and C-doped MgO and magnified 5-fold for Si- and Ti-doped MgO. For the pristine MgO surface, the DOS shows weak interactions between CO_2_ and the surface atoms. There is a slight overlapping of the minor C peak from CO_2_ with the O peak from MgO at −3.4 eV, suggesting minimal bonding contributions. An additional overlap occurs at −1.6 eV between the O peak of CO_2_ and the Mg peak of the surface, confirming weak physisorption of CO_2_ with no significant orbital hybridization. The minimal interaction aligns with the low CO_2_ adsorption energy observed for pristine MgO. Doping with Al leads to noticeable changes in the electronic states. A distinct Al peak appears at −6.8 eV, which strongly overlaps with the major O peak of CO_2_. This overlap indicates strong orbital interactions and hybridization between the Al dopant and O atom of CO_2_. Additionally, an overlap between the O peak of CO_2_ and the Mg peak at −3.9 eV further enhances the interaction. The improved adsorption energy in the Al-doped MgO can thus be attributed to the strong Al–O hybridization due to the overlap at −6.8 eV. C doping introduces a unique electronic signature in the DOS. A sharp peak at 2.5 eV overlaps with a minor O peak of CO_2_, indicating significant interaction between the C dopant and the CO_2_ molecule. Overlaps occur at multiple lower-energy positions, i.e., −5.8 eV between the C dopant and O (MgO), −4.8 eV of the minor C peak of CO_2_ overlapping with the surface O peak, −2.4 eV of the O peak of CO_2_ interacting with the Mg peak. The multiple overlaps demonstrate that C doping enhances the interaction between the C of CO_2_ and O of the MgO surface and the O of CO_2_ and Mg of the surface. Significant changes are observed in the DOS of Si-doped MgO. A dominant Si peak appears at −7.6 eV, strongly overlapping with the major O peak of CO_2_. This strong hybridization suggests a robust Si–O interaction, which significantly enhances CO_2_ adsorption. An additional interaction occurs at −5.7 eV, where the minor O peak of CO_2_ overlaps with the surface Mg peak. The Si–O orbital hybridization (strong overlap at −7.6 eV) contributes to the improved adsorption energy in Si-doped MgO. Ti doping exhibits the most pronounced electronic interactions. A dominant Ti peak appears at −0.2 eV, overlapping with the major O peak of CO_2_. This indicates strong orbital hybridization and the formation of a new Mg–O bond, which stabilizes the adsorbed CO_2_ molecule. An additional overlap occurs at −0.6 eV, where the O peak of CO_2_ interacts with the Mg peak, further enhancing stability. Therefore, the strong Ti–O hybridization, characterized by the overlap at −0.2 eV, along with the formation of new Mg–O bonds, as revealed by the DOS analysis, leads to the exceptional adsorption energy observed for Ti-doped MgO. This substantial electron polarization and bond formation make Ti-doped MgO the most effective surface for CO_2_ adsorption.

The charge transfer capacity of each dopant compared with that of Mg in pristine MgO is presented in Table 4. To understand the gain and loss of electrons in the adsorbed CO_2_ molecules, the net charges of CO_2_ and its two components, O_1_ and O_2_, are also summarized in Table 4. The minus sign indicates the acceptance of electrons. In pristine MgO, the charge transfer from the surface to the CO_2_ molecule is relatively weak. The CO_2_ molecule accepts a moderate total charge of −0.50 e^−^, distributed equally across the two O atoms (−0.63 e^−^ for O_1_ and O_2_). This symmetric charge distribution indicates a weak physisorption mechanism, where no significant electron redistribution occurs to destabilize the CO_2_ molecule. The Al in Al-doped MgO introduces a noticeable increase in the charge transfer capacity compared with pristine MgO. CO_2_ accepts −0.71 e^−^ overall, which is higher than pristine MgO. However, the charge distribution across the two O atoms remains symmetric (O_1_: −0.65 e^−^ and O_2_: −0.68 e^−^). The symmetric electron distribution indicates that no significant polarization occurs in the CO_2_ molecule. C-doped MgO exhibits the highest charge transfer capacity among the dopants, substantially increasing the electron density on the MgO surface. Although CO_2_ does not accept significantly more electrons compared with pristine MgO, the electrons donated by C are redistributed to surface atoms, increasing the surface basicity (more O^2−^ sites) and enhancing the interaction with CO_2_. Moreover, the slight charge asymmetry in O_1_ and O_2_ reflects minor polarization of the CO_2_ molecule, contributing to moderate CO_2_ stabilization. Si contributes significant charge transfer to the surface, surpassing Al but falling below C. CO_2_ accepts −1.18 e^−^, which is the highest among all dopants; however, the charge distribution is slightly asymmetric (O_1_: −0.59 e^−^ and O_2_: −0.60 e^−^). Ti provides substantial charge transfer to the MgO surface, although lower than C or Si. CO_2_ accepts −0.91 e^−^ overall. However, the charge distribution is highly asymmetric (O_1_: −0.20 e^−^ and O_2_: −0.88 e^−^). The uneven electron distribution between O_1_ and O_2_ (charge difference of 0.68 e^−^) strongly polarizes the CO_2_ molecule. This polarization induces partial breakage of the CO_2_ bond, leading to a chemically activated state and the formation of new Mg–O bonds. The strong interaction between Ti and CO_2_ is further evidenced by DOS analysis, where strong Ti–O hybridization and new Mg–O bonds contribute to the exceptional adsorption energy. Ti doping achieves the highest CO_2_ adsorption energy due to strong hybridization, significant charge transfer, and pronounced CO_2_ polarization. It is observed that the degree of polarization (difference between O_1_ and O_2_ charges) correlates directly with adsorption energy. Ti-doped MgO exhibits the highest charge difference (0.68 e^−^) and adsorption energy. Our results also show that donation electrons to the MgO surface to increase surface basic O^2−^ sites is beneficial to promote CO_2_ adsorption. This analysis aligns with previous studies (Hu et al.) that emphasize the role of dopant valence in improving CO_2_ adsorption performance [21,22]. Our findings on Ti-doped MgO suggest that transition metal elements with high valence electrons are promising candidates for further study.

### 3.4. Characterization of Pristine and C-Doped MgO

**Figure 3:**
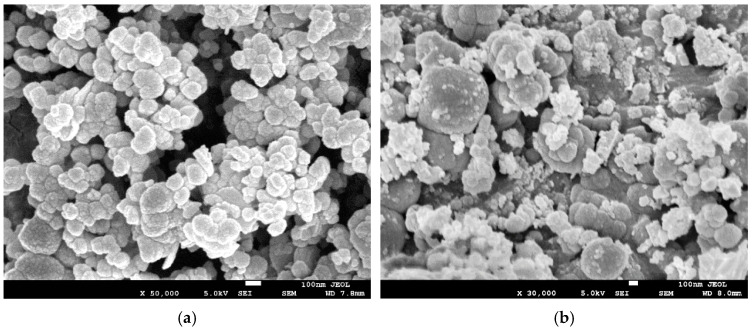
SEM images of spray-dried pristine MgO nanoparticles (**a**) and C-doped MgO nanoparticles (**b**).

Figure 3 presents scanning electron microscopy (SEM) images of pristine and C-doped MgO nanoparticles, both of which show a spherical morphology. The pristine MgO nanoparticles displayed uniformity, with an average size of around 100 nm, whereas C-doped MgO nanoparticles exhibited a broader size distribution, ranging from around 50 nm to 1 µm. Our observations are in line with previous studies, which reported changes in particle size distribution in carbon-doped materials compared with their pristine counterparts [46,47].

**Figure 4:**
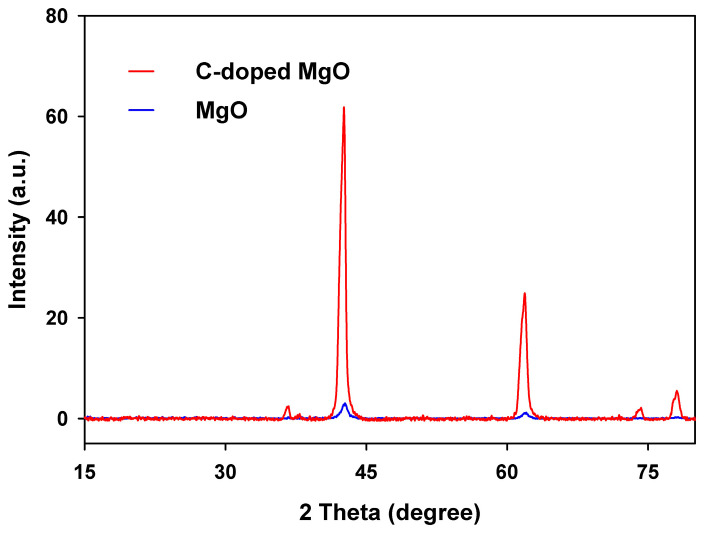
XRD spectra of MgO and C-doped MgO nanoparticles.

The structural analysis of the MgO and C-MgO were carried out by XRD analysis. Figure 4 presents the XRD spectra of the two samples, showing 2θ peaks at 36.7°, 42.6°, 62.0°, 74.2°, and 78.2°, corresponding to the (111), (200), (220), (311), and (222) lattice planes, respectively. These peaks are consistent with the standard MgO pattern (ICDD 00-045-0946). We highlight that our theoretical calculations employed identical lattice parameters for all structures, both pristine and doped. This approach is validated by the XRD spectra, which demonstrate consistent lattice parameters and structural symmetry for C-doped MgO compared with pristine MgO. Moreover, the broader full width at half maximum (FWHM) observed in the XRD peaks for C-doped MgO supports our conclusion that C doping introduces defects. These defects arise from the displacement of the doped C atom from its original Mg lattice site, as shown in Figure 2c. The carbon content in the C-doped MgO sample was determined using CHNS analysis and measured to be 5.07%.

### 3.5. Measured CO_2_ Adsorption on Pristine and C-Doped MgO

**Figure 5:**
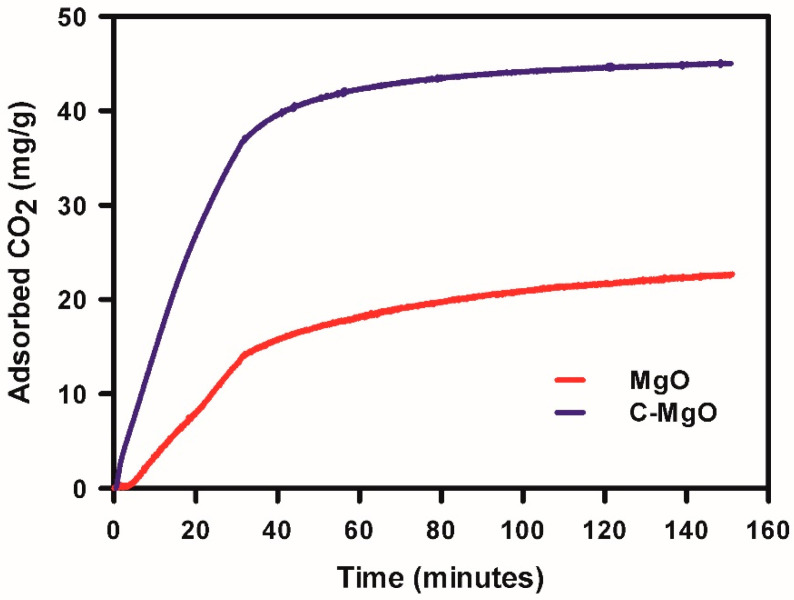
CO_2_ adsorption capacity of MgO and C-doped MgO.

The CO_2_ adsorption capacity was determined using thermogravimetric analysis. The increase in sample weight, i.e., the adsorbed CO_2_ amount, is recorded over time under a CO_2_ gas feed, as depicted in Figure 5 for pristine and C-doped MgO, respectively. It is evident that carbon doping has significantly increased CO_2_ adsorption, effectively doubling it compared with pristine MgO. Specifically, the CO_2_ capacity of the pristine MgO sample is 22.7 mg/g, whereas the C-doped MgO sample exhibits a remarkable capacity of 45.0 mg/g. The C-doped MgO shows higher CO_2_ adsorption capacity compared with the reported values of MgO doped with Zn (33.3 mg/g) [4], Al (34.1 mg/g) [12], and Cu (34.6 mg/g) [14].

## 4. Conclusions

In conclusion, while significant efforts have been dedicated to enhancing CO_2_ adsorption in MgO through the introduction of dopants, the persistent absence of explicit dopant selection guidelines and a comprehensive understanding of the underlying mechanisms remain ongoing challenges. Our investigation employed a synergistic approach that combines first-principles calculations with experimentation to explore the structural modifications in MgO doped with high-valence elements (Al, C, Si, and Ti) and their interactions with CO_2_. Structural analysis revealed that the dopants induced notable changes in bond lengths and surface geometry, particularly with Ti and C showing significant displacements. The doping formation energy indicated that Al doping is thermodynamically more favorable, while C doping is the toughest due to its higher energy requirement. CO_2_ adsorption studies demonstrated that all dopants enhanced the CO_2_ adsorption energy compared with pristine MgO, with Ti and C doping exhibiting the most pronounced improvements. Our findings uncovered two distinct mechanisms that govern CO_2_ capture: Ti-induced catalytic CO_2_ decomposition and CO_2_ polarization driven by Al, C, and Si. Specifically, Ti doping prompted structural distortion and outward displacement of Ti atoms, facilitating CO_2_ dissociation, while C doping significantly increased the electron donation capacity and CO_2_ adsorption energy. In the case of pristine and C-doped MgO, CO_2_ engagement predominantly occurred through interactions with surface O atoms, whereas Al-, Si-, and Ti-doped MgO relied primarily on interactions between the dopant and O atoms.

Experimental validation through CO_2_ adsorption measurements confirmed the superior performance of C-doped MgO, achieving a remarkable CO_2_ uptake of 45.0 mg/g—double that of pristine MgO and surpassing previously reported doped systems. This enhanced adsorption can be attributed to the increased charge donation capacity and surface basicity introduced by C doping, which facilitate stronger CO_2_ interactions.

Overall, our findings demonstrate that selective doping of MgO with high-charge-transfer elements such as C and Ti effectively enhances CO_2_ adsorption performance. These results provide profound insights into the mechanisms and principles for dopant selection and design. Future studies should explore the role of transition metal dopants and their potential for synergistic effects in CO_2_ capture and conversion technologies.

## Figures and Tables

**Table 1:** Doping formation energy and charge transfer capacity of various dopants in doped MgO.

Dopant	E_f_, eV	Charge Transfer Capacity
- *		0.54
Al	1.24	1.30
C	9.07	3.70
Si	3.64	3.37
Ti	2.04	2.54

* “-” indicates the absence of a dopant in pristine MgO. This notation is consistently applied in the subsequent Tables.

**Table 2:** Initial and optimized configuration of CO_2_ adsorption on MgO and MgO doped with Al, C, Si, and Ti.

Dopant	Initial Configuration	Most Stable Structure
-	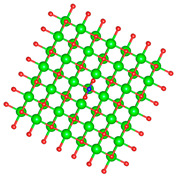	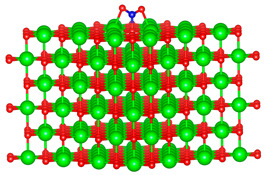
Al	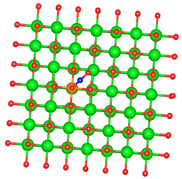	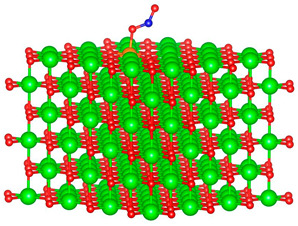
C	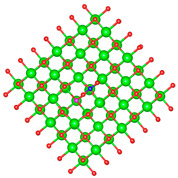	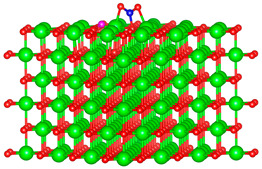
Si	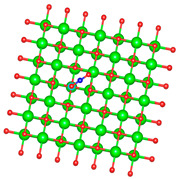	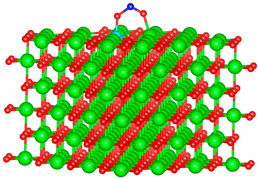
Ti	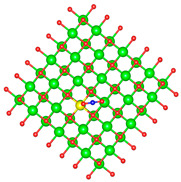	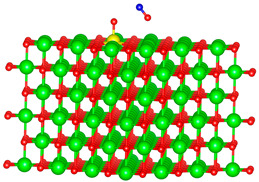

**Table 3:** Adsorption energy and structural parameters of absorbed CO_2_ molecule.

Dopant	E_ad_	Distance, Å	CO_2_ Molecule		
			<OCO, deg	d_C-O_ *, Å	d_C-O_ *, Å
-	−0.72	1.44	132.61	1.26	1.26
Al	−0.99	1.83	134.00	1.24	1.26
C	−2.77	1.42	129.35	1.26	1.27
Si	−1.39	1.63	116.37	1.27	1.42
Ti	−2.80	n/a	n/a	n/a	n/a

* d_C-O_ denotes C-O bond length.

**Table 4:** Charge transfer capacity of dopant and charge acceptance of CO_2_ molecules in CO_2_-adsorbed MgO.

Dopant	Charge Transfer Capacity	CO_2_	O_1_	O_2_
-	0.56	−0.50	−0.63	−0.63
Al	1.29	−0.71	−0.52	−0.52
C	3.79	−0.53	−0.65	−0.68
Si	2.37	−1.18	−0.59	−0.60
Ti	2.00	−0.91	−0.20	−0.88

## Data Availability

The data presented in this study are available upon request from the corresponding author.

## Supplementary Materials

The following supporting information can be downloaded at: https://www.mdpi.com/article/10.3390/biomimetics10010009/s1, Figure S1: Typical adsorption sites of doped MgO; Table S1: Procedure for simulating CO_2_ adsorption on doped MgO (100) surfaces.
