# Supplementary material for: Enhancing CO2 Adsorption on MgO: Insights into Dopant Selection and Mechanistic Pathways
Source: Biomimetics (Basel). 2024 Dec 27;10(1):9. doi: 10.3390/biomimetics10010009 (PMC11759181; doi:10.3390/biomimetics10010009)
Supplement: Supplementary file 1 [file biomimetics-10-00009-s001.zip › biomimetics-3367174-supplementary.pdf]

Enhancing CO_2 Adsorption on MgO: Insights into Dopant Selection and Mechanistic Pathways

Shunnian Wu [1], W. P. Cathie Lee [1], Hashan N. Thenuwara [1], Xu Li [2,*] and Ping Wu [1,*]

[1] Entropic Interface Group, Engineering Product Development, Singapore University of Technology and Design, 8 Somapah Road, Singapore 487372, Singapore
[2] Institute of Materials Research and Engineering, Agency for Science, Technology and Research (A*STAR), Fusionopolis Way, Innovis, Singapore 138634, Singapore
* Correspondence: x-li@imre.a-star.edu.sg (X.L.); wuping@sutd.edu.sg (P.W.)

1. Calculation procedure

Table S1 lists the 8 steps to theoretically simulate CO2 adsorption on MgO surfaces [1].

Table S1. Procedure for simulating CO_2 adsorption on MgO (100) surfaces.

Steps	Contents
1.1	**Bulk MgO optimization**
	Perform geometry optimization of bulk MgO to determine the lattice parameters.
1.2	**Build MgO slab**
	Select MgO (100) surface to build the MgO slab using Material Studios as it is the most stable surface for MgO.
	Add a vacuum layer of around 20 Å to avoid interaction between periodic images.
1.3	**Slab relaxation**
	Relax the MgO slab to allow surface atoms to adjust to their lowest energy configuration.
1.4	**Place CO_2 on the Surface**
	Position the CO_2 molecule at different adsorption sites to identify potential adsorption configurations, initially oriented perpendicular or parallel to the surface with 2.5 Å from the surface.
1.5	**CO_2/MgO system relaxation**
	Run relaxation for the CO_2/MgO system to obtain the total energy.
1.6	**Convergence Tests**
	Test for convergence of cutoff energy, k-point mesh, and slab thickness.
1.7	**Electronic structure analysis**
	Analyze the density of states (DOS) to understand changes in the electronic structure due to CO_2 adsorption.
	Perform charge analysis to quantify charge transfer between the MgO surface and CO_2 molecule.
1.8	**Post-Processing**
	Analyze adsorption energies, structural changes, electronic properties, and charge transfer to draw insights into CO_2 adsorption mechanisms on MgO.

2. Typical adsorption sites

We considered the following typical adsorption sites,[2-4] i.e., on-top site of O, on-top site of dopant, bridging site of Mg-O bond, bridging site of dopant-O bond and hole site, which have been evaluated in our preliminary work.

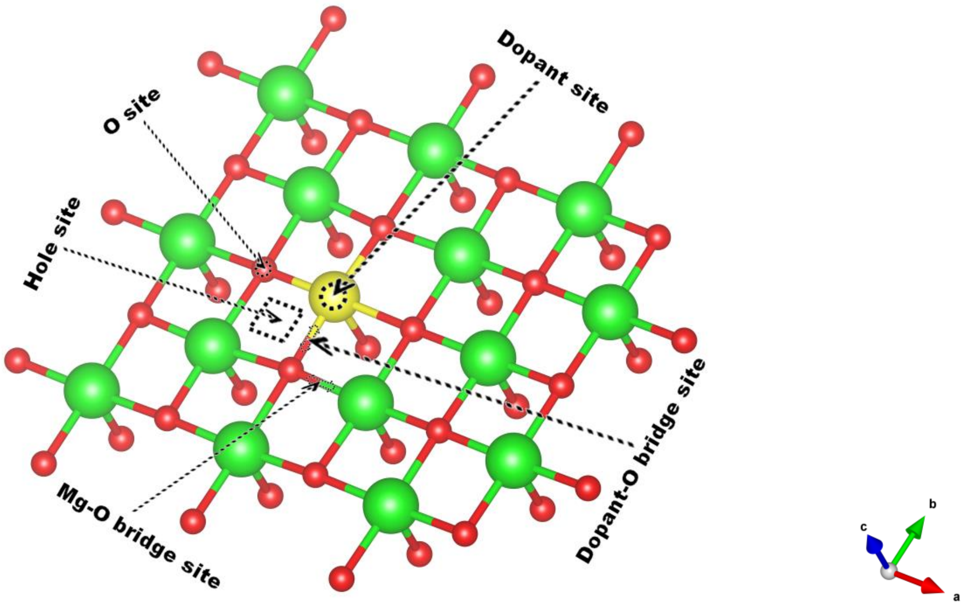

Figure S1. Typical adsorption sites of doped MgO.

1. Hafner, J. Ab-initio simulations of materials using VASP: Density-functional theory and beyond. *Journal of Computational Chemistry* **2008**, *29*, 2044-2078, doi:https://doi.org/10.1002/jcc.21057.
2. Hao, X.; Wang, B.; Wang, Q.; Zhang, R.; Li, D. Insight into both coverage and surface structure dependent CO adsorption and activation on different Ni surfaces from DFT and atomistic thermodynamics. *Physical Chemistry Chemical Physics* **2016**, *18*, 17606-17618, doi:10.1039/C6CP01689H.
3. Tosoni, S.; Spinnato, D.; Pacchioni, G. DFT Study of CO_2 Activation on Doped and Ultrathin MgO Films. *Journal of Physical Chemistry C* **2015**, *119*, 27594-27602, doi:10.1021/acs.jpcc.5b10130.
4. Ranjan, P.; Saptal, V.B.; Bera, J.K. Recent Advances in Carbon Dioxide Adsorption, Activation and Hydrogenation to Methanol using Transition Metal Carbides. *ChemSusChem* **2022**, *15*, e202201183, doi:https://doi.org/10.1002/cssc.202201183.
